# Experimental benznidazole treatment of *Trypanosoma cruzi* II strains isolated from children of the Jequitinhonha Valley, Minas Gerais, Brazil, with Chagas disease

**DOI:** 10.1590/0074-02760140260

**Published:** 2015-02

**Authors:** Jaquelline Carla Valamiel de Oliveira-Silva, Girley Francisco Machado-de-Assis, Maykon Tavares Oliveira, Nívia Carolina Noguieira Paiva, Márcio Sobreira Silva Araújo, Cláudia Martins Carneiro, Olindo Assis Martins-Filho, Helen Rodrigues Martins, Marta de Lana

**Affiliations:** 1 Núcleo de Pesquisas em Ciências Biológicas, Instituto de Ciências Exatas e Biológicas; 2 Instituto René Rachou-Fiocruz, Belo Horizonte, MG, Brasil; 3 Departamento de Farmácia, Faculdade de Ciências Biológicas e da Saúde, Universidade Federal dos Vales do Jequitinhonha e Mucuri, Teófilo Otoni, MG, Brasil; 4 Departamento de Análises Clínicas, Escola de Farmácia, Universidade Federal de Ouro Preto, Ouro Preto, MG, Brasil

**Keywords:** *Trypanosoma cruzi *II, benznidazole response, acute and chronic phases, murine model, Jequitinhonha Valley, MG, Brazil

## Abstract

Trypanosoma cruzi strains from distinct geographic areas show differences in drug resistance and association between parasites genetic and treatment response has been observed. Considering that benznidazole (BZ) can reduce the parasite burden and tissues damage, even in not cured animals and individuals, the goal is to assess the drug response to BZ of T. cruzi II strains isolated from children of the Jequitinhonha Valley, state of Minas Gerais, Brazil, before treatment. Mice infected and treated with BZ in both phases of infection were compared with the untreated and evaluated by fresh blood examination, haemoculture, polymerase chain reaction, conventional (ELISA) and non-conventional (FC-ALTA) serologies. In mice treated in the acute phase, a significant decrease in parasitaemia was observed for all strains. Positive parasitological and/or serological tests in animals treated during the acute and chronic (95.1-100%) phases showed that most of the strains were BZ resistant. However, beneficial effect was demonstrated because significant reduction (p < 0.05%) and/or suppression of parasitaemia was observed in mice infected with all strains (acute phase), associated to reduction/elimination of inflammation and fibrosis for two/eight strains. BZ offered some benefit, even in not cured animals, what suggest that BZ use may be recommended at least for recent chronic infection of the studied region.

Chagas disease, caused by the protozoan parasite *Trypanosoma cruzi,* is an important tropical disease that affects 10 million people worldwide. Most infections occur in Latin America, where this disease is endemic. It is estimated that over 10,000 people die per year due to the clinical manifestations of Chagas disease, which mainly affects the heart and the gastrointestinal tract (WHO 2010 ).

Benznidazole (BZ) is the only drug available for the specific treatment of human Chagas disease in Brazil (MS/SVS 2005). Differences in drug susceptibility of *T. cruzi* strains obtained from different geographic areas have been experimentally determined (Schlemper Jr 1982, Andrade et al. 1985 , 1989, 1992, Filardi & Brener 1987, Toledo et al. 1997 , 2002, 2003, Teston et al. 2013). The presence of *T. cruzi* strains that are naturally resistant to BZ and nifurtimox (NFX) (Filardi & Brener 1987, Andrade et al. 1992, Toledo et al. 1997) is an important fact that explains the low cure rates observed in the majority of treated patients.

It is well established that *T. cruzi* is a complex taxon that exhibits great genetic diversity. *T. cruzi* is distributed in six (I-VI) discrete taxonomic units (DTUs) (Zingales et al. 2009 ) that show significant differences related to their ecological and geographic distributions (Zingales et al. 2012). Moreover, several studies have experimentally demonstrated a significant link between the genetic diversity of *T. cruzi* strains and their biological properties (Andrade & Magalhães 1977, de Lana et al. 1998 , Revollo et al. 1998, Toledo et al. 2002), including susceptibility to chemotherapeutic agents (Andrade et al. 1985, 1989, Filardi & Brener 1987, Toledo et al. 2003) in human and experimental conditions (Andrade et al. 1992).

As the presence of parasites is essential for initiating and maintaining the pathogenic process, it is important to verify the capacity of BZ to eradicate parasites from tissues (Brener 1962, Toledo et al. 1997, 2004, Garcia et al. 2005). Studies in mice have shown that in addition to reducing parasite burden, BZ therapy can also reduce tissue damage (Andrade et al. 1989, 1991, Higuchi et al. 1993, Segura et al. 1994, Toledo et al. 1997, 2004, Garcia et al. 2005).

The drug susceptibility of* T. cruzi* strains isolated from patients with different clinical forms of the disease has been evaluated (Andrade et al. 1985, 1992, Filardi & Brener 1987, Toledo et al. 1997, Oliveira-Silva 2013). These studies can help clinicians determine when it is appropriate to treat patients with Chagas disease (Andrade et al. 1985), which was the main purpose of the Benznidazole Evaluation for Interrupting Trypanosomiasis (BENEFIT) project (Marin-Neto et al. 2009).

Our team has studied Chagas disease in the municipalities of Berilo and José Gonçalves de Minas, located in the Jequitinhonha Valley, state of Minas Gerais (MG), Brazil, which is considered to be one of the most important endemic areas of this disease in our country (Dias et al. 1985). The study location can be considered representative of the central and southern regions of Brazil, where the majority of patients are infected with parasites of the *Tc*II DTU or equivalent nomenclature (Schlemper Jr 1982, Carneiro et al. 1991, Silva et al. 2013). In this area, we have revealed the occurrence of a considerable contingent of individuals with confirmed diagnosis of Chagas disease displaying different clinical manifestations [cardiopathy, megaesophagus and megacolon, associated or not, as described by Montoya (1998), even in treated patients (de Lana et al. 2009, Machado-de-Assis et al. 2012)]. As no in vivo data exist regarding the experimental susceptibility or resistance to chemotherapeutic agents of the *T. cruzi* strains in these municipalities and that these characteristics may range even within the same *T. cruzi* DTU or genetic group or clones originating from the same *T. cruzi* strain (Camandaroba et al. 2003), the goal of the present work was to evaluate the experimental efficacy of BZ in the acute and chronic phases of infection in mice infected with the *T. cruzi* *II* strains that are predominant in this region (Oliveira-Silva 2013). The strains were isolated from children of the Jequitinhonha Valley before treatment with BZ and they are still under evaluation.

## SUBJECTS, MATERIALS AND METHODS

*Patients and T. cruzi strains - *Eight *T. cruzi* strains (501, 795, 806, 817, 829, 855, 1661 and 2405) isolated from children with a recent chronic infection by haemoculture (Hm) prior to treatment (Filardi & Brener 1987) were evaluated. Six strains were identified in a serological investigation of Chagas disease in Berilo and José Gonçalves de Minas, two municipalities that are in close proximity (Borges et al. 2006). Subsequently, two other strains isolated from children before treatment were included in the study. All strains were classified as *T. c*ruzi II by analysing the polymorphisms of the isoenzymes of cytochrome oxidase subunit II, the mini-exon intergenic regions and 24Sα rDNA genes of *T. cruzi*. All strains presented low virulence in mice (Oliveira-Silva 2013) and were biologically homogenous.

*Animals and experimental T. cruzi infection* - Female Swiss mice (28-30 days old) obtained from the Animal Science Centre of the Federal University of Ouro Preto (UFOP), MG, were used for the study. For each *T. cruzi* strain, four groups of eight mice were inoculated intraperitoneally with 1.0 x 10^4^ blood trypomastigotes per animal. The inoculum was determined as described by Brener (1962).

*Treatment schedule* - BZ treatment was performed in the acute phase (TAP) and chronic phase (TCP) of infection, starting at 10 and 90 days after inoculation, respectively. Animals were treated only after detection of parasites by fresh blood examination (FBE) or Hm. Groups of eight animals were treated daily with BZ (Rochagan^(r)^; LAFEPE, Brazil), 100 mg/kg of body weight for 20 consecutive days. For treatment, BZ tablets were resuspended in Arabic gum and administered by gavage.

*Evaluation of mice during and after treatment* - All mice of the TAP group were evaluated in parallel to the untreated control group as follows.

*FBE* - Five microlitres of blood collected from the tail vein of mice were examined for parasitaemia on alternate days from the fourth day after infection (Brener 1962). The patent period (PP), maximum peak of parasitaemia (MPP) and day of MPP (DMPP) were also determined for each strain. FBE was performed to confirm the presence of infection before the start of treatment and, subsequently, to assess reactivation of parasitaemia after treatment. The results were expressed as the percentage of mice with positive FBE. This examination was used only in the acute phase of infection.

*Hm* - Thirty days after the end of TAP and TCP, mice that were negative for parasitaemia by FBE were submitted to Hm according to Filardi and Brener (1987). Each culture tube was examined for the presence of parasites after 30, 60, 90 and 120 days and the results are expressed as the percentage of mice with positive Hm for each strain in the treated and non-treated (NT) groups. Hm was also employed to confirm infection in mice with subpatent parasitaemia.

*Polymerase chain reaction (PCR) of peripheral blood - *Blood samples were collected from the retro-orbital plexus of mice with negative FBE and Hm results at 30 days after treatment. The samples were mixed in a 1:1 proportion with 6 M guanidine/0.2 M EDTA, pH 8.0 and were stored at room temperature (Ávila et al. 1991). DNA extraction was performed according to a modified Gomes et al. (1998) method. PCR amplifications were carried out using S35 and S36 primers (Ávila et al. 1991) to amplify a specific fragment of 330 base pairs of kinetoplast DNA of *T. cruzi*. The reaction mixture was submitted to 35 amplification cycles with a thermocycler (PTC-150; MJ Research). Amplified DNA was visualised in silver-stained 6% polyacrylamide gels. The percentage of mice with positive PCR results was obtained for each strain in the treated and NT groups.

*Conventional serology (CS)-ELISA* - This test was performed according to Voller et al. (1976). Samples of sera were collected six months after the end of the TAP and TCP and stored at -20ºC. Sera were tested at 1:80 dilutions in phosphate-buffered saline using an antigen of the *T. cruzi* Y strain prepared by alkaline extraction of parasites obtained during exponential growth in LIT medium. Antibody binding was detected using peroxidase-labelled anti-mouse immunoglobulin G antibody (Sigma Immunochemical Reagents, USA). The absorbance was read in a spectrophotometer with a 490-nm filter (model 3550; Bio-Rad). The cut-off value was calculated for each plate considering the mean absorbance of 10 negative control serum samples plus two standard deviations (SD). The percentage of mice with positive ELISA was obtained for each strain in the treated and NT groups.

*Non-CS [flow cytometry for the detection of anti-live trypomastigote antibodies (FC-ALTA)]* - The FC-ALTA was performed using the same samples evaluated by ELISA, according to the Cordeiro et al. (2001) adaptation to microplates of the method of Martins-Filho et al. (1995). Sera from the experimental animals were assayed at 1:1.500 and 1:3.000 dilutions using goat anti-mouse IgG antibody (Sigma Immunochemical Reagents) labelled with fluorescein isothiocyanate (FITC) to assess IgG reactivity. The results were expressed as the percentage of positive fluorescent parasites (PPFP) based on the internal control of non-specific binding of the FITC-conjugated second-step reagent. Positive and negative controls were included in all experimental batches. Flow cytometric measurements were performed on the FACSCalibur flow cytometer (Becton-Dickinson, USA). Samples were considered negative when PPFP was ≤ 20% and positive when PPFP was > 20%, as described by Martins-Filho et al. (1995). The percentage of mice with positive FC-ALTA was obtained for each strain in the treated and NT groups.

For animals treated at the TCP of infection, the same methodologies employed for those in the TAP of infection were used, except FBE, due to the low parasitaemia of the animals.

*Cure criterion* - Drug susceptibility and resistance were defined by the cure criterion based on parasitological or molecular parasitological (FBE, Hm and PCR), CS (CS-ELISA) and non-CS (FC-ALTA). Animals were grouped as follows: treated not cured (TNC) animals, animals with at least one positive parasitological test and/or at least one positive serological test, dissociated animals with negative results in all parasitological tests (FBE/Hm/blood PCR) and FC-ALTA, but with positive CS-ELISA, and treated cured (TC) animals with negative results in all parasitological and serological tests.

*Drug resistance and susceptibility criterion* - The cure rates were calculated by determining the ratio (number of mice cured/total number of mice, TAP or TCP) x 100. To determine the in vivo susceptibility of *T. cruzi* strains to BZ, the following classifications were used: resistant (cure rates ≤ 33%), partially susceptible (cure rates > 33% - < 67%) and susceptible (cure rates ≥ 67%), according to Toledo et al. (2003).

*Histopathological analysis - *Three treated and untreated animals per group (TAP, TCP) were necropsied at the TCP of infection (180 days after treatment) to verify if the treatment with BZ in the TAP and TCP prevents lesions in the heart during the course of the infection. The heart was fixed in 10% buffered formalin (pH 7.2) and embedded in paraffin. Sections of 5 µm thickness were mounted on glass slides and stained with haematoxylin and eosin. The morphometric studies of inflammation involved analysing images of 15 randomly-selected fields (total area 1.15 × 10^6 ^µm^2)^ of tissue sections for a single slide per animal. Inflammatory infiltration of the heart was quantified by counting the cell nuclei. The inflammatory process was determined by the difference (p < 0.05) between the number of cell nuclei present in the heart of animals infected with *T. cruzi* and the number observed in uninfected animals ± SD (Caliari 1997, Maltos et al. 2004). Images taken with a 40X objective were analysed with Leica QWin software (Leica Microsystems, Germany).

*Statistical analysis* - Data for biological parameters (PP, PM, DPMP and area under the curve of parasitaemia) were analysed using the program Prism 5 for Windows, v.5.0. The Kolmogorov-Smirnov test of normality was used for data corresponding to all parameters. Data with normal distributions were evaluated by ANOVA followed by the Newman-Keuls post-test. For data not normally distributed, the nonparametric Mann Whitney *U* test was employed. The analysis of mortality and infectivity was carried out using the chi-squared test. The comparison of the mean number of inflammatory cells in the untreated group and the treated group was performed by the nonparametric Mann Whitney *U* test. Differences were considered statistically significant at p ≤ 0.05, with a confidence interval of 95%.

*Ethics* - The inclusion of patients in the study and the blood collections were performed after obtaining a signed consent form that was approved by the Ethical Committee for Research in Humans from the René Rachou Research Centre, Oswaldo Cruz Foundation, Belo Horizonte, MG (process 007/02) following the Helsinki Declaration of 1975 revised in 2008.

The animal study was approved by the Ethical Committee in Animal Experimentation of the UFOP (process 2009/10). Animals were maintained according to the guidelines of the Brazilian School of Animal Experimentation.

## RESULTS

*Parameters evaluated in the TAP: parasitaemia curve - *In TAP animals, etiological treatment led to an important and significant reduction of parasitaemia (area under the mean curve of parasitaemia) in all strains in relation to the NT group (Table I). The parasitaemia became subpatent in animals infected with all strains, except strain 806, during treatment. In some of these mice, parasitaemia was patent up to the 16th day of treatment. Reactivation of parasitaemia was observed in animals infected with strains 795, 817, 885 and 1661 between days 14-30 after the end of treatment and between days 44-60 after infection (Table I). The parasitaemia was subpatent before, during and after treatment only in animals infected with strain 829.

**TABLE I.** Parasitaemia before and after the end of treatment and parasitaemia reactivation in mice inoculated with *Trypanosoma cruzi* strains treated with benznidazole

*T. cruzi* strains	Mean of the area under the curve of parasitaemia (TAP/INT) x (10^3^)	Day of the beginning of the TAP	Day of the end of the TAP	Parasitaemia reactivation
806	5.838.87 ± 4.950.92/1.269.51 ± 637.98^*a*^	447,000	1,500	-
817	2.227.57 ± 2.091.34/152.93 ± 65.41^*a*^	14,760	0	Yes
885	721.54 ± 645.99/65.32 ± 81.64^*a*^	4,375	0	Yes
1661	5.037.37 ± 2.612.17/217.5 ± 63.21^*a*^	28,000	0	Yes
795	1.121.18 ± 904.64/46.19 ± 114.72^*a*^	1,250	0	Yes
829	20.82 ± 18.28/0.0 ± 0.0^*a*^	0	0	-
501	1.163.70 ± 986.71/1 ± 2.64^*a*^	0	0	-
2405	563.00 ± 516.40/2.00 ± 3.46^*a*^	0	0	-

*a*: significant difference; INT: infected not treated; TAP: treated in the acute phase.

*PP, MPP and DMPP - *For all strains that showed patent parasitaemia during the TAP, a notable reduction of the PP was observed. For all strains, except 806, the mean MPP was significantly lower in animals of the TAP group in relation to the NT group (Table II). A significant reduction was observed in the mean DMPP of animals of the TAP group compared to animals of the NT group. The opposite was observed in mice infected with strain 1661, which had a higher mean DMPP in the TAP group than in the NT group, but that difference was not significant (Table III). For the majority of strains, the TAP group showed lower PP, MPP and early DMPP values than those of the NT group (Table III).

**TABLE II.** Positive results of parasitological and serological methods in mice infected with *Trypanosoma cruzi* II strains isolated from children and treated in the acute (TAP) and chronic (TCP) phases of infection

	TAP n/n (%)				TCP n/n (%)		
*T. cruzi* strains	Parasitological methods (FBE, Hm, PCR)	Serological methods (ELISA, FC-ALTA)	Cure		Parasitological methods (Hm, PCR)	Serological methods (ELISA, FC-ALTA)	Cure
501	5/8 (62.5)	1/8 (12.5)	3/8 (37.5)		8/8 (100)	8/8 (100)	0/8 (0)
795	7/7 (100)	7/7 (100)	0/7 (0)		4/7 (57)	7/7 (100)	0/7 (0)
806	8/8 (100)	7/7 (100)	0/8 (0)		3/5 (60)	5/5 (100)	0/5 (0)
817	7/7 (100)	6/7 (85.7)	0/7 (0)		4/4 (100)	4/4 (100)	0/4 (0)
829	8/8 (100)	8/8 (100)	0/8 (0)		5/7 (71.4)	5/5 (100)	0/7 (0
855	8/8 (100)	7/7 (100)	0/8 (0)		3/6 (50)	5/5 (100)	0/6 (0)
1661	8/8 (100)	8/8 (100)	0/8 (0)		6/8 (75)	8/8 (100)	0/8 (0)
2405	8/8 (100)	7/8 (87.5)	0/8 (0)		6/7 (85.7)	6/6 (100)	0/7 (0)
Total	59/62 (95.1)	51/60 (85)	3/62 (4.8)		39/52 (75)	48/48 (100)	0/52 (0)

FBE: fresh blood examination; FC-ALTA: flow cytometry for the detection of anti-live trypomastigote antibodies; Hm: haemoculture; PCR: polymerase chain reaction.

**TABLE III.** Mean of biological parameters evaluated in mice infected and treated in the acute phase (TAP) and infected not treated (INT) during the acute phase with strains of *Trypanosoma cruzi* isolated from children

	Patent period			Maximum peak of parasitaemia			Day of maximum peak of parasitaemia	
*T. cruzi* strains	TAP/INT	p		TAP	p		TAP x INT	p
501	0 ± 0/25 ± 4.0	0.002		0 ± 0/122.5 ± 43.4	0.002		0 ± 0/21 ± 3.5	0.000
795	0.28 ± 0.2/34.5 ± 3.5	0.007		1.42 ± 0.9/68.6 ± 17.1	0.000		2.85 ± 1.8/21.5 ± 2.4	0.013
806	8 ± 0.9/20.2 ± 1.8	0.000		447 ± 93.5/840.9 ± 364.4	0.645^*a*^		10 ± 0/15.75 ± 2.2	0.009
817	8 ± 0.7/26.25 ± 3.1	0.000		29.9 ± 4.6/238.2 ± 84.6	0.027		12.75 ± 0.5/23.5 ± 0.8	0.000
829	0 ± 0/3.3 ± 1.1	0.009		0 ± 0/ 5.5 ± 1.4	0.009		0 ± 0/18 ± 4.1	0.008
855	3.5 ± 1.3/21.5 ± 3.8	0.000		10 ± 3.5/98.1 ± 31.7	0.015		8.5 ± 2.6/18.2 ± 0.8	0.002
1661	7.25 ± 1.4/27.2 ± 1.2	0.000		31 ± 5.1/471.7 ± 96.8	0.000		24 ± 9.0/17.7 ± 0.8	0.100^*a*^
2405	0.25 ± 0.2/23.25 ± 5.8	0.003		1 ± 0.6/58 ± 16.7	0.003		4 ± 2.6/26.25 ± 4.9	0.003

*a*: no significant difference.

*Cure control* - Considering all parasitological and molecular parasitological methods (FBE, Hm and PCR), 95.1% of the animals in the TAP group showed positive results (59/62) (Table II). These methods detected therapeutic failure in 100% of the animals infected with strains 795, 806, 817, 829, 855, 1661 and 2405 (Table II). For mice infected with strain 501, 37.5% showed negative results (3/8). For the NT animals, 100% of the FBE and Hm results were positive (57/57) (unpublished observations). The parasitological and molecular parasitological methods (Hm and PCR) showed that 75% (39/52) of TCP animals showed positive results, ranging from 50-100% between the different infected groups (Table II). For the NT group, 93.1% (54/58) of animals had positive parasitological results and for the different groups infected with each *T. cruzi* strain, the positive parasitological results ranged from 75-100% (unpublished observations).

Considering both serological methods (ELISA and FC-ALTA), 85.5% (51/60) of the animals in the TAP group showed positive results (Table II). For both the TAP and TCP subgroups of the NT group, 100% (62/62 and 51/51, respectively) showed positive serological results (data not shown). For the TCP group, the serological methods were positive in 100% of the animals (48/48) (Table II); this was also the case for the NT group (data not shown).

*Index of cure - *Following the established cure criterion, 95.1% (59/62) of the animals in the TAP group were considered TNC and only 4.8% (3/62) of the animals infected with strain 501 were considered parasitologically cured (Table II). All animals (52/52) treated at the TCP of infection were TNC.

*Response to BZ - *According to the classification of drug resistance/susceptibility used in this study, strain 501 was partially susceptible to BZ because the index of cure observed in mice in the TAP group was 37.5% (3/8). Animals infected with the other strains were not cured by treatment with BZ during the TAP and all strains were considered resistant to treatment at this phase of the infection. In the TCP group, no animal was cured after treatment and all strains were considered 100% resistant to BZ at the TCP of infection.

*Histopathology* - In relation to Figure (A, B), it is important to clarify that the same pattern of NT inflammation was used for the TAP and TCP groups infected by the 501 and 806 strains because the aspect of both were very similar.

In the TAP animals, the histopathological evaluation revealed that only mice infected with strain 501 displayed a lower number of inflammatory cells than the control NT group (A, B in Figure). Interestingly, the inflammation in mice infected with this strain remained even in TC animals. In TAP mice infected with strain 806, no inflammation was observed, which is different from the corresponding NT group (A, B in Figure).

In TCP mice infected with all *T. cruzi* strains, no inflammation was observed (A, B in Figure). Fibrosis was detected only in animals infected with strain 806 (C in Figure). Fibrosis was more intense in the NT group (C2 in Figure), but was still present in animals treated in the TAP and TCP (C3, 4, respectively, in Figure). Treated and NT animals infected with strains other than 806 in both the TAP and TCP of infection did not show neoformation of collagen.

## DISCUSSION

Several authors have demonstrated differences in the therapeutic response to etiological treatment of patients with Chagas disease (Coura & de Castro 2002). This work demonstrated the BZ resistance profile of *T. cruzi* DTU II strains isolated from infected children before treatment. This was verified in a murine model during the experimental TAP and TCP of infection.

The results obtained from mice that were treated with BZ during the TAP of infection showed a significant reduction in the parameters related to parasitaemia (area under the curve of parasitaemia, PP and MPP) in relation to the group infected, but NT. This corroborates the results obtained by other studies in mice (Toledo et al. 2004, Caldas et al. 2008, Olivieri et al. 2010, Teston et al. 2013). Additionally, the resistance profiles of the *T. cruzi* DTU II strains to BZ in mice treated in the TAP and TCP of infection were similar. However, our experimental results corroborate the low cure rate observed in humans from distinct areas of the central region of Brazil and MG (Ferreira 1990, Braga et al. 2000, Lauria-Pires et al. 2000), a fact that is concerning due to the great number of people with Chagas disease requiring treatment in the Jequitinhonha Valley, including the children from whom our strains were isolated. Only strain 501 was partially susceptible to BZ in the TAP. These results are discordant with those of Toledo et al. (2003), who studied clonal stocks of *T. cruzi *of different genotypes, including *T. cruzi* II (equivalent to genotype 32), and obtained a cure rate of 80% and 69.2% in mice treated in the ATP and TCP, respectively. Here, as in Toledo et al. (2003), for seven out of eight strains, relative homogeneity in biological behaviour and drug response were observed (Silva et al. 2013). The difference in the drug response of *T. cruzi *II strains studied here (all resistant to BZ) and those studied by Toledo et al. (2003), which were partially sensitive or sensitive, could be due to the different geographical origins of the parasite populations used in the two studies. Here, the strains used were from two municipalities that are only 13 km from each other (Berilo or José Gonçalves de Minas). In the study by Toledo et al. (2003), the strains used were from Brazil, Chile and Bolivia. On the other hand, Filardi and Brener (1987) also verified that strains isolated from MG showed great variability in BZ and NFX susceptibility/resistance in mice. More recently, Campos et al. (2005) studied five clones of the 21SF strain, all of which were *T. cruzi* II strains, in Swiss mice treated with BZ; they found cure rates of 30-100%, while the percentage of cure with the parental strain was 25%. This further suggests that the variability of drug response observed in clonal populations of *T. cruzi* II strains is responsible for the high variation of results obtained with BZ and NFX chemotherapy observed in strains of this biodeme.

**Figure:**
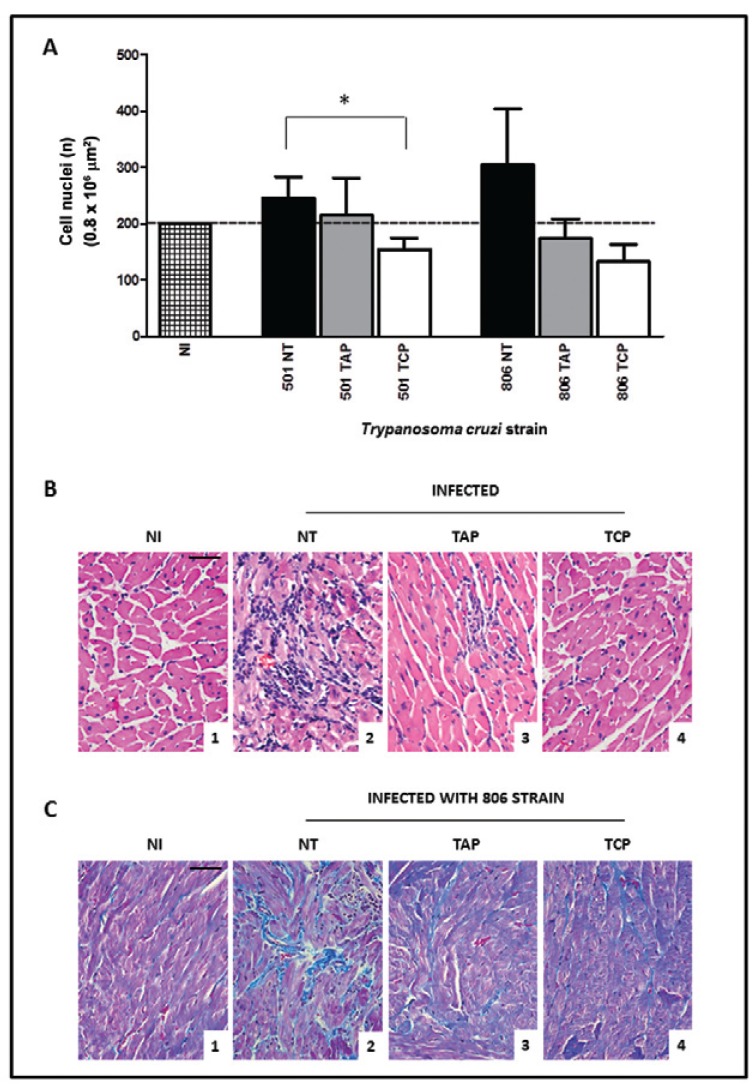
A: number of nuclei cells in the heart of mice infected with 501 and 806 *Trypanosoma cruzi* strains isolated from children of Berilo, Jequitinhonha Valley, state of Minas Gerais, Brazil ( : discrete; : moderate; : intense). The asterisk means significant difference; B: representative photomicrographs of inflammatory process [1: normal histological appearance in uninfected animals (NI); 2: inflammatory infiltrate in the heart of not treated (NT) mice infected with 501 or 806 *T. cruzi* strains; 3: rare focus of inflammation in mice infected with 501 or 806 strains treated in the acute phase (TAP) of the infection; 4: normal aspect of the histological preparation of mice infected with 501 or 806 strains treated in the chronic phase (TCP) of the infection. Haematoxylin and eosin. Bar = 50 µm]; C: representative photomicrographs of fibrosis process (1: normal histological appearance in NI animals; 2: mice infected with 806 *T. cruzi* strain and NT; 3: TAP; 4: collagen deposition in mice infected with 806 *T. cruzi* strain and in the TCP of the infection. Trichrome Masson. Bar = 50 µm).

The pos-treatment evaluation using parasitological tests (FBE, Hm and PCR) showed that 95.2% (59/62) of animals treated during the TAP and 75% (39/52) of animals treated during the TCP were positive, respectively. PCR was more sensitive for the detection of therapeutic failure compared with FBE and Hm, as demonstrated in other studies in mice (Caldas et al. 2008, Miyamoto et al. 2008) and dogs (Guedes et al. 2002).

Three mice infected with strain 501 and treated during the TAP of infection presented negative results in parasitological tests and negative results in ELISA (CS) and FC-ALTA (non-CS); thus, they were considered to be the only mice that were parasitologically cured, according to the criterion of cure adopted in this study. Thus, strain 501 revealed an index of cure of 37.5% (3/8); so, we classified this strain as partially resistant to BZ. The therapeutic failure of treatment in the TAP of infection, as indicated by positive parasitological results, was confirmed by positive serological results in the majority of animals.

All animals (13/52) that were treated during the TCP and were parasitologically negative were positive by both serological tests (6 months after treatment) and were thus considered not cured. The therapeutic failure of the animals treated in the TCP was 100%, as confirmed by the serological tests used. Based on these results, all strains (100%) were considered to be resistant to BZ when treated in the TCP of infection in this murine model.

After treatment, the histopathological analysis of the TAP groups compared to the NT groups did not show inflammation in the hearts of the animals, except in those infected with strain 501. The histopathological analysis of the cured mice infected with this strain showed discrete inflammation, which was different from the not cured animals, who showed moderate inflammation. The opposite was observed in animals infected with strain 806 because BZ treatment eliminated the inflammation (Figure) that was normally observed in the NT.

This result suggests that even in the mice that were not cured, BZ treatment reduced and prevented the occurrence of at least this type of lesion in mice infected with the 501 and 806 strains. Three TAP animals (3/8) infected with strain 501 reduced the inflammation, which was discrete when compared to the NT group. BZ treatment benefits were also observed in mice infected with the 806 strain in both phases of infection, which was similar to the results of previous studies in mice (Andrade et al. 1991, Toledo et al. 1997, 2004, Olivieri et al. 2010). However, our results did not correspond with those of Caldas et al. (2008) in mice infected with AAS and VL-10 strains (*Tc*II lineage, resistant to BZ).

Fibrosis was observed only in mice infected with strain 806: it was most intense in the NT group, less intense in the TAP group and even less intense in the TCP group, which suggests that the reduction in parasitism after BZ treatment may also reduce heart damage. This finding is consistent with the results of Caldas et al. (2008) in mice infected with strain VL-10 (*T. cruzi* II, also resistant to BZ) treated in the TAP and with the results of Portella and Andrade (2009), who observed lower cardiac inflammation and fibrosis in mice treated with BZ in the TCP of infection compared with the untreated group. Additionally, an interesting study by Andrade et al. (1991) showed that fibrosis was reversible in mice treated in the TCP although some contradictions were reported by Andrade et al. (1989) and Garcia et al. (2005) when treated and NT animals were compared.

Although a parasitological cure was not observed in animals infected and TCP, the beneficial effects of BZ were also verified in these animals, because no inflammation was observed in all experimental groups, except in those animals infected with 501 and 806 strains. Even with some contradictions, these results, together with others observed in humans (Gallerano & Sosa 2000, Streiger et al. 2004, Souza-Estani & Segura 2006, Viotti et al. 2014), suggest that the etiological treatment of Chagas disease should be performed for all serologically positive individuals in the TAP of disease and those in the chronic indeterminate phase and with non-advanced or benign clinical forms of chronic Chagas disease (MS/SVS 2005 **).** Additionally, an important project named BENEFIT is in progress with the objective of verifying the real effect of the etiological treatment of chronic patients with the cardiac form of the disease (Marin-Neto et al. 2009). In fact, human treatment still remains a great challenge and studies with the objective of discovering new drugs that are more safe and effective for both phases of the infection are urgently needed (Coura & de Castro 2002, Romanha et al. 2010).

In conclusion, despite the resistance of the majority (7/8) of *T. cruzi* strains in mice, a beneficial effect of treatment was partially demonstrated because reduction and/or suppression of parasitaemia were observed during the treatment of animals in the TAP of infection infected with all strains. This reduction was associated with a reduction or elimination of inflammation (except in animals infected with strain 501) and fibrosis was intense only in the mice infected with strain 806 treated in both phases of infection.

Even though we cannot extrapolate results obtained in animals to humans, these results support the recommendation of treatment for people with a confirmed diagnosis of Chagas disease in the two municipalities examined in this study in the Jequitinhonha Valley. It is likely that treatment should be recommended in other areas where *Tc*II DTU of *T. cruzi* is predominant. Currently, we are following the effect of BZ treatment in the children from whom these strains were isolated before treatment. In the two last evaluations, one of the children was serologically negative. However, more evaluations in the coming years are necessary for better comparisons and conclusions regarding experimental and human BZ treatment efficacy. Additionally, we hope that our results will stimulate other researchers to perform similar studies in other regions of our country and Latin America to expand on the results of our study and further support etiological treatment administration to generate hope for the people infected with Chagas disease in poor endemic regions.
